# Blue Lunula Related with Hydroxyurea

**DOI:** 10.4274/tjh.2011.0016

**Published:** 2013-03-05

**Authors:** Hava Üsküdar Teke, Abdülsamet Erden

**Affiliations:** 1 Eskişehir Osmangazi University, Faculty of Medicine, Department of Hematology, Eskişehir, Turkey; 2 Kayseri Education and Research Hospital, Internal Medicine, Kayseri, Turkey

**Keywords:** Neoplasia, Hematopoiesis

Hydroxyurea, which is an inhibitor of ribonucleoside reductase and is used as a systemic antitumor agent, is frequently used for the treatment of myeloproliferative and hematologic diseases. While the most common side effect is myelosuppression, gastrointestinal symptoms such as nausea, vomiting, stomatitis, and anorexia and rash, skin ulcers, hyperpigmentation in nails, and dermatologic toxicities like facial erythema may also be observed. At higher dosages, neurologic symptoms may be rarely seen (1,2). 

A 76-year-old female patient applied to the hematology polyclinic in August 2010 complaining about weakness. She had no other diseases except for hypertension. She had used acetylsalicylic acid (ASA) and an angiotensin receptor antagonist. In her examinations it was found that her white blood cell count was 13.7 x 103/µL, hemoglobin level was 14.8 g/dL, mean corpuscular volume was 60 fL, and platelet level was 1161 x 103/µL. Among biochemical parameters, her potassium level was 5.9 meq/L, lactate dehydrogenase was 319 IU/L, sedimentation was 12 mm/h, ferritin was 22.9 µg/L and fibrinogen was normal, and C-reactive protein was normal. In her physical examination, it was seen that her liver was 1-2 cm palpable under the costa, Traube’s space was closed, and the spleen was 1-2 cm palpable under the costa. The thrombocytes were increased in the peripheral blood smear and defective thrombocytes were present. Her history revealed that her thrombocyte level was 787 x 103/µL in 2008 and 1097 x 103/µL in 2009. Bone marrow aspiration and biopsy were carried out considering essential thrombocythemia. The JAK-2 V617F gene mutation was determined to be positive and Ph chromosome was determined to be negative. Her bone marrow biopsy was observed as hypercellular and megakaryocytes were observed, including multilobular nucleotides, which increased in number and were generated as clusters. After the essential thrombocythemia diagnosis, the patient was treated with hydroxyurea (1000 mg/day). It was also recommended that she continue using ASA. 

The patient came for follow-up after 3 weeks. In her physical examination, it was seen that blue pigment changes were present in the lunula regions of the nails of both hands and feet (see Figure 1). It was determined that the PT-PTT-INR-fibrinogen level was normal, hemoglobin level was 13.6 g/dL, platelet count was 583 x 103/µL, and white blood cell count was 8.1 x 103/µL. It was found that the pigment changes in the nails of the patient were caused by hydroxyurea; use of hydroxyurea was stopped and replaced by use of anagrelide. When the patient came for examination after 2 further weeks, her nail lesions were decreased, and after the fourth week, these lesions had completely disappeared. 

It is known that hydroxyurea, which is a ribonucleoside reductase inhibitor and is used as a systemic antitumor agent in hematologic diseases, causes brown pigmentation in nails. However, blue lunula related with hydroxyurea is rarely observed (2,3). In our study the blue color change (lunular pigmentation) occurred in both hand and foot lunula regions 3 weeks after the start of the treatment, and 4 weeks after the cessation of hydroxyurea, the color returned to normal. 

**Conflict of Interest Statement**

The authors of this paper have no conflicts of interest, including specific financial interests, relationships, and/ or affiliations relevant to the subject matter or materials included.

## Figures and Tables

**Figure 1:**
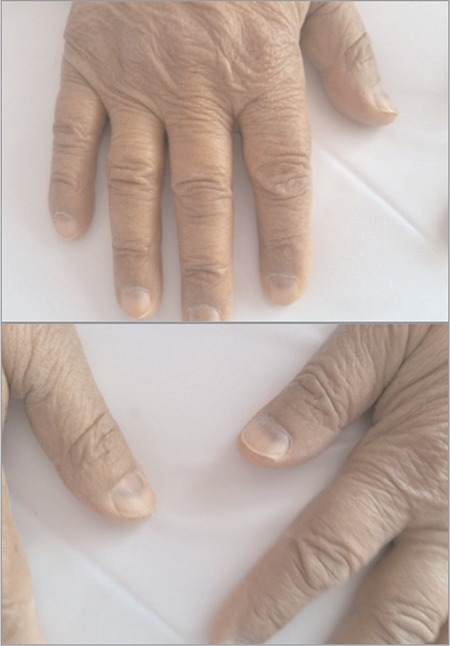
Blue pigment changes in the lunula regions of hand nails
